# Membrane Curvature and Cancer: Mechanisms, Implications, and Therapeutic Perspectives

**DOI:** 10.3390/cancers18071076

**Published:** 2026-03-26

**Authors:** Alexandros Damalas, Ioannis D. Kyriazis, Marijonas Tutkus, Charalampos Angelidis, Varvara Trachana

**Affiliations:** 1 Department of Biology, Faculty of Medicine, University of Thessaly, Biopolis, 41500 Larissa, Greece; damalas@uth.gr (A.D.); ioankyriazis@uth.gr (I.D.K.); 2 Institute of Biotechnology, Life Sciences Center, Vilnius University, LT-10257 Vilnius, Lithuania; marijonas.tutkus@gmc.vu.lt; 3 Department of Molecular Compound Physics, Center for Physical Sciences and Technology, LT-10257 Vilnius, Lithuania; 4 Department of Biology, Faculty of Medicine, School of Health Sciences, University of Ioannina, 45110 Ioannina, Greece; chaggeli@uoi.gr

**Keywords:** membrane curvature (MC), cancer, Ras, EMT, caveolae, endocytosis

## Abstract

Membrane curvature is a fundamental biophysical property of cellular membranes that underlies essential processes such as vesicle formation, organelle shaping, intracellular trafficking, and membrane scission. While traditionally studied in the context of cell biology and membrane dynamics, membrane curvature is now emerging as a critical, albeit underrecognized, regulator of oncogenic transformation and tumor progression. Curvature not only governs the mechanical properties of the membrane but also influences the spatial localization and activation of key signaling proteins, including Ras family GTPases, whose oncogenic functions are closely dependent on membrane topology. Cancer is frequently associated with disruptions in the regulation of membrane curvature as a result of aberrant lipid metabolism, overexpression of curvature-modulating proteins, and cytoskeletal remodeling. These changes facilitate the hallmarks of malignancy such as uncontrolled proliferation, enhanced motility, immune evasion, metabolic rewiring, and therapy resistance. Notably, recent evidence reveals that curvature acts as a spatial cue for Ras activation, particularly during epithelial-to-mesenchymal transition (EMT), where curvature-driven Ras relocalization amplifies growth factor signaling and promotes metastasis. This review provides a comprehensive overview of the molecular determinants that generate and sense membrane curvature from lipid shape and membrane asymmetry, BAR domain proteins, and actin dynamics, and explores how these mechanisms are hijacked in cancer. We describe the feedback between membrane architecture and oncogenic pathways such as Ras/MAPK and PI3K/AKT, emphasizing the role of curvature in shaping signal transduction platforms. It should be noted that “curvature-driven signaling” is defined as signaling regulation that arises from membrane-geometry-dependent localization, clustering, or activation of signaling proteins, while “curvature-sensitive platforms” refer to membrane subdomains whose specific curvature selectively recruits and stabilizes signaling complexes. Furthermore, we examine how these biophysical alterations impact vesicular trafficking, organelle morphology, and secretion, all of which are co-opted to support tumor development. From a translational standpoint, we assess emerging therapeutic strategies designed to target curvature-regulating factors and leverage membrane topology for precision drug delivery. Innovations in nanomedicine, super-resolution imaging, and curvature-sensing biosensors are also discussed as tools for both diagnostics and therapeutic monitoring. By integrating advances in membrane biophysics, cancer signaling, and bioengineering, this review highlights membrane curvature as a central and actionable dimension of cancer biology.

## Simple Summary

Cancer research has traditionally focused on genetic mutations and biochemical signaling pathways, but growing evidence suggests that the physical properties of cell membranes also play an important role in tumor development. One such property is membrane curvature, which refers to how the cell membrane bends and changes shape during processes such as vesicle formation, cell movement, and intracellular transport. Furthermore, recent studies indicate that abnormal membrane curvature can influence the localization and activity of key cancer-related proteins, including Ras, thereby affecting signaling pathways that control cell growth, survival, and metastasis. This review highlights how changes in membrane shape arise from alterations in lipids, proteins, and the cytoskeleton in cancer cells, and how these changes help tumors progress. By summarizing current knowledge and emerging technologies, this work aims to encourage the research community to consider membrane curvature as an important regulatory factor and a potential target for future cancer therapies.

## 1. Introduction

While membrane curvature has been extensively reviewed in the context of endocytosis, cytoskeletal remodeling, and BAR-domain protein function, these studies have largely treated curvature as a downstream structural consequence of cellular activity. The present review advances a distinct conceptual framework by positioning membrane curvature as an active, upstream biophysical regulator of oncogenic signaling and cancer cell state transitions. Rather than cataloguing curvature-associated proteins in isolation, we highlight evidence showing how curvature dynamically integrates lipid metabolism, cytoskeletal forces, vesicular trafficking, and signal transduction into spatially organized signaling platforms that cancer cells exploit. In particularly, we emphasize membrane curvature as a physical determinant of oncogenic Ras localization, nanoclustering, and pathway activation, including during epithelial–mesenchymal transition, thereby linking membrane architecture directly to cancer signaling logic. By reframing curvature as a regulatory layer that coordinates biochemical and mechanical cues across multiple cancer hallmarks, this review provides an integrative perspective that goes beyond prior descriptive accounts and highlights membrane curvature as a unifying and actionable principle in cancer biology.

Cell membranes are complex, dynamic structures composed of lipids, proteins, and carbohydrates that together form a selectively permeable barrier crucial for cellular integrity and function. Beyond serving as a passive boundary, membranes are active participants in virtually every aspect of cell physiology, including signal transduction, intracellular transport, energy conversion, and intercellular communication. A critical and often underappreciated property of these membranes is their curvature—defined as the local bending or deformation of the lipid bilayer. This curvature is not static but is dynamically regulated to accommodate processes such as vesicle budding, membrane fission and fusion, endocytosis, exocytosis, and the formation of specialized subcellular structures like filopodia and lamellipodia.

In this review, membrane curvature is treated not as a purely qualitative geometric feature, but as a continuous biophysical parameter that integrates membrane geometry, mechanical forces, and energetic constraints. Curvature arises from defined physical determinants, including lipid asymmetry, protein scaffolding or insertion, cytoskeletal forces, membrane tension, and protein crowding, and is dynamically regulated across spatial scales. Importantly, specific curvature regimes can selectively modulate protein localization and signaling efficiency. In cancer, dysregulation of these parameters reshapes membrane curvature landscapes, thereby promoting aberrant signaling, trafficking, and invasive cell behavior.

Membrane curvature is not merely a geometric characteristic; it is a biophysical signal that governs and responds to a wide range of cellular cues [1]. Certain proteins—such as those containing Bin/Amphiphysin/Rvs (BAR) domains—and lipids with intrinsic curvature properties act as curvature sensors and inducers, helping the membrane adapt to functional demands. Importantly, changes in curvature often coincide with biochemical signaling events, suggesting that cells interpret curvature as a spatial code that guides molecular localization and activity. For example, the distribution and activation of small GTPases, such as Ras, which require precise membrane anchoring to exert their oncogenic functions, are increasingly understood to be regulated by curvature-driven compartmentalization [2].

In the context of oncogenesis, these processes become dysregulated. Cancer cells often exhibit alterations in lipid metabolism, cytoskeletal dynamics, and expression of curvature-modulating proteins, all of which converge to distort membrane architecture. The distortions are not incidental, but are functionally significant, enabling cancer cells to internalize and recycle growth factor receptors, resist apoptosis, reorganize the cytoskeleton for migration, and secrete pro-tumorigenic factors via vesicles. Moreover, there are recent reports that membrane curvature can directly influence the localization and activation of oncogenic signaling pathways, including PI3K/AKT, MAPK, and notably Ras. For instance, epithelial-to-mesenchymal transition (EMT), a process critical for invasion and metastasis, has been shown to involve curvature-dependent recruitment of Ras to the plasma membrane in response to TGFβ stimulation [3,4].

Despite its emerging importance, membrane curvature remains a relatively underexplored dimension of cancer biology. Most cancer-focused studies emphasize genetic and biochemical alterations, while overlooking the physical and mechanical properties of cells that are equally vital to disease progression. However, understanding how cancer cells sense, generate, and exploit membrane curvature may reveal new mechanisms of malignancy and open avenues for therapeutic intervention. This is particularly pertinent as technologies such as super-resolution microscopy, curvature-sensitive biosensors, and curvature-responsive drug delivery systems become more accessible and sophisticated.

This review aims to provide a comprehensive overview of membrane curvature in cancer. We begin by outlining the molecular mechanisms that generate and sense membrane curvature, followed by an examination of how curvature modulates key hallmarks of cancer such as vesicle trafficking, receptor signaling, organelle morphology, and motility. We then explore how these processes are co-opted by oncogenic pathways—especially Ras—and conclude by discussing emerging therapeutic opportunities that exploit curvature-based vulnerabilities. By integrating biophysical principles with cancer signaling biology, we aim to establish membrane curvature as a central and actionable component of tumor development and treatment [5].

Cellular studies are uniquely suited to reveal where and when curvature-dependent processes occur within the complex intracellular environment. These approaches have been instrumental in defining the spatial and temporal localization of BAR-domain proteins, Ras nanoclusters, and endocytic machinery, while integrating the contributions of cytoskeletal forces, lipid metabolism, and signaling feedback loops [6,7]. Such observations establish clear physiological relevance, yet they are often limited in their ability to disentangle direct effects of membrane curvature from confounding biochemical and mechanical variables inherent to living cells.

In contrast, in vitro reconstitution systems—including liposomes, supported lipid bilayers, membrane nanotubes, and microfabricated substrates—offer precise control over membrane composition, curvature radius, and tension, enabling direct interrogation of curvature sensing and generation mechanisms [8,9,10]. These reductionist platforms have been critical for demonstrating curvature-dependent binding affinities of BAR-domain proteins, amphipathic helices, and lipid-anchored signaling proteins such as Ras, and for extracting quantitative parameters that inform and refine cellular models [5,7].

Importantly, progress in the field increasingly depends on an iterative dialogue between these experimental strategies. Hypotheses generated from cellular imaging and perturbation studies are tested and parameterized in controlled in vitro systems, while predictions emerging from reconstitution experiments are subsequently validated in living cells and higher-order models, including organoids and microfluidic platforms. This convergence has proven particularly powerful in elucidating links between membrane curvature and oncogenic signaling, endocytosis, and mechanotransduction, where in vitro approaches provide mechanistic clarity and cellular studies establish functional and disease relevance [11].

Together, these advances underscore that cellular and in vitro approaches should not be viewed as competing alternatives but rather as mutually reinforcing strategies. Their integration is essential for achieving a comprehensive and mechanistically grounded understanding of curvature-dependent regulation in cancer biology.

## 2. Mechanisms of Membrane Curvature Generation

Disrupting membrane curvature—via hypoosmotic shock or caveolae perturbation—releases Ras from the membrane, thereby blunting signaling. This finding demonstrates that membrane biophysics can directly regulate oncogenic signaling strength and duration [4,5].

Mechanistically, this process is dependent on the lipid-modified C-terminal hypervariable region of Ras, which senses membrane architecture. Ras membrane localization is essential for GTP-loading and downstream activation. This reveals a novel feedback loop: TGFβ signaling induces curvature that enhances Ras activation, which in turn sustains TGFβ/SMAD signaling, driving EMT and metastatic progression [4].

**Figure 1:**
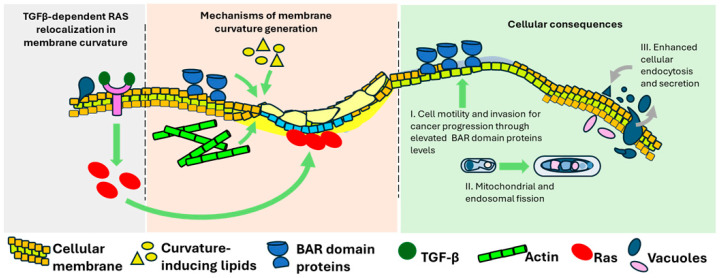
Membrane Curvature and Cancer: Mechanisms, Implications, and Therapeutic Perspectives. This schematic illustrates the relationship between membrane curvature and cancer biology. (**Left**): TGFβ signaling induces membrane curvature followed by Ras relocalization, linking extracellular cues to intracellular oncogenic pathways. (**Middle**): Membrane curvature can be generated through alterations in lipid composition and asymmetry, curvature-sensing and -generating proteins like BAR domain proteins, and cytoskeletal contributions such as actin dynamics. (**Right**): In cancer cells, membrane curvature influences critical functions including endocytosis, secretion, organelle morphology, mitochondrial and endosomal fission. Additionally, BAR domain proteins serve as characteristic hallmark of cell motility and invasion serving a distinct cancer cellular consequence.

Recent experimental findings by Damalas et al. (2022) [4] have elucidated a compelling mechanism linking TGFβ-induced membrane curvature changes to Ras oncoprotein plasma membrane localization during EMT. In breast cancer cells, TGFβ-1 treatment significantly increases positive membrane curvature, which is detected by curvature-sensing proteins and coincides with the relocalization of H-ras and, to a lesser extent, K-ras to the plasma membrane [4,7] (Figure 1).

Beyond facilitating membrane relocalization, membrane curvature can directly tune oncogenic signaling output. Curved membrane domains promote Ras nanocluster formation, modify local lipid environments, and stabilize Ras–effector interactions, thereby enhancing signaling strength and prolonging pathway activation. Moreover, curvature-dependent compartmentalization at specific plasma membrane or endosomal sites can bias downstream effector engagement, contributing to signaling specificity rather than acting solely as a spatial recruitment mechanism [7,12,13].

### 2.1. Lipid Composition and Asymmetry

Membrane curvature can arise intrinsically from lipid composition and transbilayer asymmetry. Lipids with distinct molecular geometries impose bending stresses on the bilayer, with cone-shaped lipids favoring negative curvature, inverted cone-shaped lipids promoting positive curvature, and cylindrical lipids stabilizing flat membranes, as described in classic membrane biophysics studies by McMahon and Gallop 2005 [6] and Parton and Simons 2007 [14].

Asymmetric lipid distribution between membrane leaflets generates spontaneous curvature through differential packing stress and is maintained by lipid translocases, as outlined by McMahon and Gallop 2005 [6]. In cancer, altered lipid metabolism reshapes membrane composition and asymmetry, enriching membranes in curvature-prone lipid species [15,16,17]. These lipid-driven effects establish the basal curvature landscape upon which protein-mediated curvature sensing and generation act. Lipids are the primary structural components of membranes, and their shape strongly influences membrane curvature. Cone-shaped lipids such as phosphatidylethanolamine (PE) and cardiolipin promote negative curvature, while inverted cone-shaped lipids like lysophosphatidylcholine (LPC) favor positive curvature. Cylindrical lipids such as phosphatidylcholine (PC) stabilize flat bilayers [18,19,20]. The asymmetric distribution of these lipids between the inner and outer membrane leaflets introduces spontaneous curvature.

### 2.2. Curvature-Sensing and -Generating Proteins

Proteins that sense or induce curvature are fundamental to membrane remodeling. Chief among these are the Bin/Amphiphysin/Rvs (BAR) domain proteins, which recognize and stabilize curved membrane regions (Figure 1). Proteins, such as amphiphysin, endophilin, FCHo [21,22], and CIP4 [23,24], engage membranes through electrostatic and hydrophobic interactions. Membrane curvature can be generated through two principal mechanisms: (1) the insertion of amphipathic helices or hydrophobic protein motifs into one leaflet of the lipid bilayer, which creates local asymmetry and deforms the membrane, and (2) the introduction of structural scaffolding by proteins with intrinsically curved domains (such as BAR, F-BAR, or ENTH domains), which impose their shape onto the membrane surface to stabilize and propagate curvature [25,26]. Moreover, altered expression or activity of FBP17 [27] and CIP4 in breast and colorectal cancer cells, respectively, has been shown to promote invadopodia [28,29], suggesting that these proteins can modulate membrane curvature dynamics with direct consequences for cancer cell invasion. However, their primary mechanistic role remains the physical modulation of membrane shape to facilitate processes like vesicle formation and cytoskeletal coordination [30]. Such curvature-sensing proteins have been show to regulate the localization of signaling complexes, including Ras nanoclusters, which preferentially localize to curved, lipid raft-rich domains to enhance signaling amplitude [31], thereby providing spatial precision to oncogenic signals [5].

Beyond the well-characterized BAR domain family, membrane curvature is orchestrated by multiple protein classes that operate at different spatial scales and within different membrane compartments. ENTH- and ANTH-domain-containing proteins, such as epsins, couple phosphoinositide binding to clathrin coat assembly and actively promote membrane bending during endocytosis, linking curvature generation to receptor internalization and signaling regulation [32,33,34,35].

Dynamin family GTPases further contribute by stabilizing highly curved membrane necks and driving scission, while also acting as curvature sensors whose activity is modulated by membrane tension and lipid composition [36].

Caveolins and cavins constitute another major curvature-regulating system, organizing caveolae as specialized invaginations that buffer membrane tension and compartmentalize oncogenic signaling pathways [37,38]; disruption of this system alters mechanotransduction and growth factor signaling in cancer cells of intracellular organelles, reticulons and DP1/Yop1 [39,40].

There is also evidence that mechanical stress from the extracellular matrix (ECM) or neighboring cells can induce curvature changes that activate mechano-transductive pathways, including those involving YAP/TAZ and Ras [41].

### 2.3. Cytoskeletal Contributions to Curvature

The cytoskeleton, particularly actin filaments and their regulatory proteins, plays a key mechanical role in generating membrane curvature (Figure 1). Actin polymerization produces protrusive forces that deform the plasma membrane, forming structures such as lamellipodia and filopodia. This is initiated by nucleators like the Arp2/3 complex and formins, which define filament architecture. Actin-binding proteins such as myosin II generate contractile forces that further assist in membrane invagination, particularly during vesicle scission. These cytoskeletal processes are tightly coordinated with curvature-sensing proteins, ensuring that membrane remodeling events occur with spatial and temporal precision [15,42,43].

In cancer cells, membrane curvature emerges from a dynamic interplay between lipid-driven mechanisms and cytoskeletal forces rather than from a single dominant process. Actin-mediated forces then act on this lipid-defined landscape to actively sculpt and remodel curvature through polymerization-driven protrusion, contractility, and force transmission to the membrane [44]. In highly motile and invasive contexts—such as lamellipodia, filopodia, and invadopodia formation [45]—actin-driven forces can transiently dominate curvature generation, whereas in endocytic trafficking, organelle morphology, and oncogenic Ras nanocluster stabilization, lipid-driven curvature and membrane composition play a more prominent role. Importantly, these mechanisms are tightly coupled through bidirectional feedback loops: lipid composition regulates the recruitment and activity of actin regulators and curvature-sensing proteins, while cytoskeletal forces reciprocally influence lipid redistribution and membrane tension. Thus, rather than acting independently, lipid-driven and cytoskeletal mechanisms are best viewed as integrated components of a unified curvature-regulatory system that cancer cells exploit to spatially organize signaling platforms, sustain oncogenic signaling, and adapt membrane architecture to changing microenvironmental and mechanical demands [8].

### 2.4. Membrane Tension and Mechanical Forces

Membrane tension, which is the in-plane force within the lipid bilayer, directly influences membrane flexibility. Lower tension allows for easier deformation, while high tension resists curvature. This parameter is dynamically modulated by cellular processes including cytoskeletal rearrangement, osmotic pressure changes, and membrane trafficking. Mechanotransduction pathways integrate these mechanical cues, allowing cells to modulate curvature in response to physical stimuli [46]. At the mechanistic level, tension serves as a gatekeeper for curvature-generating events like endocytosis and filopodia formation [44,47].

This machinery is hyperactivated in cancer. For instance, overexpression of Arp2/3 and formins correlates with invasive phenotypes, and curvature-sensitive complexes promote invadopodia formation during matrix degradation and invasion [48].

Myosin II and other actin-binding proteins generate contractile forces that contribute to membrane invagination and vesicle scission. These forces are integrated with curvature-sensing domains in endocytic proteins, enabling coordination between the cytoskeleton and membrane remodeling [49].

Similarly, actin polymerization at the membrane generates pushing forces, leading to protrusions such as lamellipodia and filopodia. Nucleators like the Arp2/3 complex and formins initiate and organize these filaments, while cofilin and gelsolin modulate filament turnover [50].

### 2.5. Protein Crowding and Phase Separation

In addition to lipid composition and cytoskeletal forces, membrane curvature is also influenced by protein crowding and phase separation. High local concentrations of membrane-bound proteins generate lateral pressure, which can promote local curvature. Similarly, the formation of liquid-like domains through protein phase separation can concentrate curvature-inducing factors, such as BAR domain proteins and lipid-modifying enzymes. These microdomains create specialized regions of the membrane with distinct curvature and functional properties, enabling compartmentalization of signaling and trafficking processes [51,52].

High local concentrations of membrane-associated proteins generate lateral pressure within the bilayer, which can directly promote membrane bending [51]. Increasing evidence indicates that such crowding often arises through liquid–liquid phase separation driven by intrinsically disordered proteins (IDPs), which form dynamic, liquid-like condensates at the membrane [52]. These condensates act as organizational platforms that locally concentrate structured curvature-regulating factors, rather than deforming the membrane directly.

Within these protein-dense microdomains, curvature-inducing and curvature-sensing proteins—such as BAR-domain family members—as well as lipid-modifying enzymes become selectively enriched [51,52]. BAR-domain proteins, while not intrinsically disordered themselves, are efficiently recruited to phase-separated environments where high protein density and altered lipid composition lower the energetic barrier for membrane deformation. In this way, IDP-driven phase separation and protein crowding provide a biophysical framework that amplifies the activity of classical curvature-generating proteins.

The resulting membrane microdomains display distinct curvature and functional properties, enabling spatial compartmentalization of signaling and trafficking processes. Such coupling between protein phase behavior and membrane remodeling offers a versatile mechanism for dynamically organizing curvature-dependent signaling platforms, particularly in contexts where rapid and reversible membrane reconfiguration is required, such as endocytosis, signal transduction, and cancer-associated membrane plasticity [52].

### 2.6. Organelle-Specific Curvature Regulation

Organelles maintain a characteristic membrane curvature that is related to their function. This is achieved through the action of specialized proteins that stabilize or induce curvature. For example, the endoplasmic reticulum (ER) relies on reticulons and atlastins to maintain a tubular network, while mitochondrial fission is driven by the curvature-inducing protein DRP1. An increase in fission promotes metabolic reprogramming toward glycolysis (Warburg effect). Fission also aids mitophagy and resistance to apoptosis [53]. Similarly, dynamic changes in the curvature of the Golgi apparatus are mediated by lipid composition and cytoskeletal tension. These organelle-specific mechanisms demonstrate how curvature is tightly regulated across intracellular compartments to support diverse cellular functions [54].

## 3. Functional Roles of Membrane Curvature in Cancer

In addition to gene overexpression, epigenetic regulation and post-translational modifications (PTMs) also modulate the function of curvature-related proteins. PTMs, including palmitoylation and farnesylation, dictate the membrane localization of Ras and sensitivity to curvature. Such lipid modifications are regulated by enzymes such as farnesyltransferase (FTase) and acyl-protein thioesterases, which are themselves altered in certain cancers. Thus, curvature regulation is not only a product of genetic changes but may also be a consequence of dynamic post-translational and epigenetic programming [55].

Similarly, acetylation or phosphorylation of BAR domain proteins can alter membrane affinity and curvature-sensing capacity. Notably, histone modifications near genes encoding for curvature modulators like amphiphysin or EHD2 have been observed in breast and prostate cancer datasets, suggesting transcriptional deregulation via epigenetic means [56].

### 3.1. Endocytosis and Receptor Recycling

The flexibility of membranes is not merely a structural feature but also serves as a regulatory axis for nearly every aspect of cancer cell behavior. For example, membrane curvature affects intracellular trafficking, endocytosis, organelle dynamics, and signal transduction, all of which are hijacked in malignancy (Figure 1). Both clathrin-mediated and clathrin-independent endocytosis pathways are often upregulated in cancer cells in order to support recycling rather than degradation of growth factor receptors such as EGFR, HER2, MET, and integrins [57,58]. These receptors are responsible for transmitting mitogenic and survival signals, and their sustained recycling promotes oncogenic signaling persistence [59]. Curvature-inducing proteins, including clathrin, dynamin, and BAR domain family members, facilitate vesicle budding and scission. Internalized receptors continue to signal from endosomal compartments, where curvature helps define specialized signaling microdomains, amplifying pathways like MAPK and PI3K/AKT [60]. In this context, dynamin is frequently upregulated in aggressive tumors and facilitates scission of endocytic vesicles, including those that internalize EGFR and other mitogenic receptors [61]. Similarly, increased expression of F-BAR proteins like CIP4 enhances lamellipodia formation and is associated with poor prognosis in breast and lung cancers. Importantly, these proteins also form physical and functional interactions with small GTPases such as Ras, Rac1, and Cdc42, amplifying curvature-dependent motility and invasion [62,63].

Collectively, these observations illustrate how membrane curvature is not merely a structural feature but an active regulator of multiple cancer hallmarks. By shaping endocytosis, receptor recycling, vesicular signaling, and cytoskeletal dynamics, curvature provides a spatial and mechanical framework that sustains oncogenic signaling, promotes invasion, and supports tumor progression.

### 3.2. Exocytosis and Secretion

Curvature also plays a pivotal role in the exocytosis of vesicles, with the consequent release of signaling molecules and extracellular vesicles (Figure 1). Cancer cells exploit this mechanism to secrete matrix-degrading enzymes, cytokines, and pro-tumorigenic exosomes. Proteins such as SNAREs and Rab GTPases coordinate vesicle docking and fusion, processes that require tightly regulated membrane curvature. Elevated exosome release in Ras-driven cancers reflects the upregulation of curvature-sensitive trafficking pathways, which promote intercellular communication and metastatic niche formation [64].

Ras isoforms localize to distinct subcellular membranes during oncogenic activation. For example, recycling endosomes enriched in curvature and lipid rafts serve as hubs for KRAS nanocluster formation, enhancing MAPK and PI3K pathway activation [65].

### 3.3. Organelle Morphology and Function

As already mentioned, the shape and function of cellular organelles are closely related to membrane curvature. Morphological changes may support cancer cell proliferation, migration, and stress resistance [66]. For example, increases in mitochondrial fission mediated by DRP1, impact the metabolism and increase the resistance to cell death. Similarly, the shape of the ER adapts to meet increased protein synthesis demands, while Golgi fragmentation, which is partly curvature-dependent, enhances secretory activity [67] (Figure 1).

This increase depends on proteins like SNAREs, Rab GTPases, and curvature-modifying lipids, which coordinate vesicle docking and fusion. In Ras-driven cancers, the increase in exosome release involves vesicles loaded with oncogenic cargo, including mutant KRAS, which can be horizontally transferred to other cells, propagating malignant signaling [64].

Similarly, increases in exocytosis of matrix metalloproteinases (MMPs), cytokines, and extracellular vesicles (EVs), including exosomes and microvesicles, promote matrix degradation, immune evasion, angiogenesis, and metastatic niche formation [68].

### 3.4. Cell Motility and Invasion

Cancer cell motility involves the formation of curved plasma membrane structures such as lamellipodia, filopodia, and invadopodia. These are generated through coordinated actin polymerization and the recruitment of curvature-generating proteins like BAR domain family members (Figure 1). Curvature enables directional cell movement and extracellular matrix degradation, facilitating invasion. Small GTPases like Rac1 and Cdc42 regulate these processes downstream of oncogenic Ras, integrating curvature dynamics with cytoskeletal remodeling and signaling activation [26,69].

### 3.5. TGFβ-Induced Membrane Curvature and Ras Activation

We have previously reported that TGFβ signaling induces membrane curvature changes that facilitate the relocalization of Ras to the plasma membrane during epithelial-to-mesenchymal transition (EMT) [4] (Figure 1). This curvature-dependent Ras recruitment enhances downstream signaling, reinforcing TGFβ/SMAD activity and promoting metastatic progression. These findings reveal a feedback mechanism where extracellular cues modulate membrane shape to spatially organize oncogenic signals, with curvature serving as a physical determinant of Ras activation efficiency [4].

Small GTPases such as Rac1 and Cdc42—often downstream of Ras—activate actin nucleators like Arp2/3 to generate curvature-associated motile structures. Additionally, invadopodia formation is enhanced in cells undergoing epithelial-to-mesenchymal transition (EMT), where curvature-sensitive proteins concentrate at leading edges to promote directional invasion [11].

### 3.6. Distinct Mechanisms of Curvature-Dependent Regulation of Oncogenic Pathways

Membrane curvature can directly regulate multiple signaling pathways beyond its role in membrane trafficking, in addition to ligand-dependent activation of GPCRs—where curvature and lipid packing modulate ligand affinity, receptor oligomerization, and stabilization of active conformations [70].

Small Rho-family GTPases, such as Rac1 and Cdc42, also display curvature-dependent recruitment to membrane protrusions, where local curvature stabilizes actin-regulatory signaling complexes during cell migration and invasion [71].

These observations establish membrane curvature as an active biophysical regulator of diverse signaling systems rather than a purely permissive or trafficking-related feature. In the case of Ras, however, curvature sensitivity arises from a fundamentally distinct mechanism. Ras proteins lack transmembrane domains and rely exclusively on lipid-modified hypervariable regions for membrane association, rendering their plasma membrane recruitment, lateral diffusion, and nanocluster formation intrinsically dependent on membrane topology. As a result, Ras directly senses and transduces membrane curvature [13] into changes in signaling output, rather than responding indirectly through receptor conformational changes or cytoskeletal intermediates. This unique mode of curvature-dependent regulation positions Ras as a particularly direct interpreter of membrane architecture, a feature that becomes especially consequential during TGFβ-induced EMT, where curvature remodeling amplifies Ras localization and oncogenic signaling [4].

## 4. Molecular Dysregulation of Curvature Mechanisms in Cancer

### 4.1. Proteins That Regulate Membrane Curvature and Endocytosis

The scientific literature concerning the implication of proteins in membrane curvature and endocytosis regulation in cancer progression is rapidly increasing. Among the proteins explored, dynamin-2 expression and activity have been linked to enhanced receptor trafficking, cell migration and invasion, and tumor progression in multiple cancer types, with evidence of correlations to aggressive clinical features. Endophilin family BAR-domain proteins, which sense and induce curvature during endocytic events, also show altered expression in malignancies; for example, endophilin A1 (SH3GL1) is elevated in breast carcinoma and associates with tumor stage and metastasis. More broadly, disruptions in endocytic pathways—of which curvature-regulating proteins are key components—contribute to dysregulated signal transduction and oncogenic behaviors in cancer cells [72,73,74].

### 4.2. Altered Lipid Metabolism and Membrane Composition

In addition to the changes in protein expression already discussed, cancer cells also exhibit widespread reprogramming of lipid metabolism, resulting in membrane compositions that favor high curvature. Oncogenic signaling induces enzymes like fatty acid synthase (FASN), acetyl-CoA carboxylase (ACC), and stearoyl-CoA desaturase (SCD1), which synthesize unsaturated and cone-shaped lipids. Cancer cells also upregulate phosphatidylinositol 4,5-bisphosphate (PIP2) and phosphatidic acid (PA), which serve as docking platforms for curvature-sensing proteins and Ras signaling complexes. These lipid changes not only support vesicle formation and trafficking but also modulate membrane fluidity and stiffness. Moreover, the enrichment of lipid rafts with cholesterol and sphingolipids modifies membrane stiffness and fosters the formation of curved nanodomains that promote the clustering of Ras nanodomains and enhance signal transduction efficiency [75,76].

Altered lipid metabolism in cancer can be directly translated into membrane curvature through established biophysical mechanisms. Changes in lipid molecular shape and leaflet asymmetry—such as enrichment of cone-shaped phosphatidylethanolamine or phosphatidic acid versus inverted-cone lipids like lysophosphatidylcholine—generate spontaneous curvature, while acyl-chain saturation and cholesterol content regulate lipid packing, membrane order, and bending rigidity (κ), thereby tuning membrane stiffness and deformability [9,77]. In parallel, cancer-associated increases in anionic signaling lipids such as phosphatidylserine, PIP_2_, and phosphatidic acid alter membrane electrostatics and promote recruitment of curvature-sensing or curvature-generating proteins, amplifying lipid-driven deformation [78]. Although direct correlations between tumor lipidomic signatures and specific curvature phenotypes in patient samples remain limited, extensive evidence from reconstituted membranes and cancer cell models demonstrates that defined lipid compositions produce predictable changes in membrane mechanics and curvature-dependent processes [16].

### 4.3. Feedback with Oncogenic Pathways

Membrane curvature and oncogenic signaling are reciprocally regulated. Curved membrane regions support the spatial organization and clustering of signaling molecules such as Ras. The localization of H-ras, K-ras, and N-ras to membranes is governed by their lipid modifications and the curvature of the membrane environment. Recent findings demonstrate that positive membrane curvature facilitates the recruitment of Ras to the plasma membrane, especially during EMT triggered by TGFβ. Once localized, Ras activates a suite of downstream effectors—including RAF, PI3K/AKT, MAPK pathways, and RalGDS—that reinforce cellular transformation and metastasis. Thus, membrane curvature both influences and is influenced by oncogenic Ras signaling, forming a self-reinforcing loop that sustains malignancy [4,5]. For example, PI3K/AKT signaling enhances the production of curvature-prone lipids, while MAPK activation drives the expression of actin remodeling proteins that assist in membrane deformation [79,80].

## 5. Therapeutic Targeting of Membrane Curvature in Cancer

Interdisciplinary collaboration between biophysicists, oncologists, nanotechnologists, and computational scientists will be essential to translate membrane curvature insights into patient benefits.

From a clinical perspective, membrane curvature offers a largely untapped area that can be utilized for diagnosis, prognosis, and therapy. Biomarkers based on curvature-sensing proteins or curvature-altered vesicle profiles may enable early detection and stratification of aggressive tumors. Furthermore, as curvature-targeting drugs and nanoparticles advance, clinical trials could usefully monitor curvature status using non-invasive imaging or circulating vesicle profiles as a measure of efficacy [55,81].

### 5.1. Inhibitors of Curvature-Modulating Proteins

Given its central role in regulating membrane dynamics, signaling compartmentalization, and cell motility, membrane curvature represents a promising but underexploited therapeutic frontier in oncology. Aberrant curvature machinery in cancer cells offers multiple intervention points—including curvature-generating proteins, lipid biosynthesis pathways, and curvature-dependent trafficking systems.

Regarding proteins, dynamin inhibitors, such as dynasore and Dyngo-4a, impair clathrin-mediated endocytosis by blocking vesicle scission. This reduces internalization of oncogenic receptors like EGFR, inhibiting downstream signaling through Ras/MAPK pathways. BAR domain proteins, which are overexpressed in many cancers, are also emerging targets. Small molecules and peptides that inhibit BAR domain dimerization or membrane association can disrupt invadopodia formation and metastasis [82]. Dual-targeting strategies combining curvature-protein inhibitors with downstream pathway blockers (e.g., Rac1, Cdc42) may act synergistically to enhance therapeutic efficacy. Notably, targeting CIP4 and FBP17 suppresses metastatic behavior in breast and colon cancer models [28,83].

### 5.2. Lipid Metabolism Modulators

Altering membrane lipid composition can modulate curvature and disrupt oncogenic signaling domains. Pharmacologic agents targeting lipid biosynthesis—such as FASN inhibitors (e.g., TVB-2640) and SCD1 inhibitors—reduce the abundance of unsaturated and cone-shaped lipids that support curvature formation. Statins interfere with cholesterol metabolism and lipid raft integrity and impair Ras membrane anchoring. Farnesyltransferase inhibitors (FTIs) prevent Ras prenylation, thereby disrupting curvature-dependent signaling compartments [84,85]. These approaches offer indirect but potent opportunities to target membrane architecture in cancer.

### 5.3. Nanotechnology and Curvature-Responsive Drug Delivery

Nanotechnology provides a novel means of exploiting cancer-specific membrane curvature for targeted therapy. Because tumor cells exhibit elevated endocytic activity and altered lipid composition, they preferentially internalize nanoscale drug carriers such as liposomes, micelles, and dendrimers.

Curvature-responsive nanocarriers, including liposomes, micelles, and dendrimers, are preferentially internalized by tumor cells due to their elevated endocytic activity and unique lipid composition [86,87]. Some nanoparticles are engineered to disassemble or release their drug cargo only in the presence of specific curvature or lipid environments. For instance, pH-sensitive liposomes coated with amphipathic peptides preferentially bind to curved tumor membranes and release drugs in acidic microenvironments [88].

Curvature-sensing peptides derived from BAR domains such as modified amphiphysin helices can also be used to guide therapeutic payloads directly to tumor-associated membrane structures such as invadopodia. Furthermore, CRISPR/Cas9-loaded nanoparticles, engineered to selectively disrupt genes encoding curvature-regulating proteins (e.g., CIP4, FCHo2), offer a functional genomics approach to suppress metastasis [89].

Emerging approaches also include sphingolipid analogs and PIP2 modulators, which alter membrane charge and curvature to disrupt oncogenic signaling hubs.

Pharmacologic agents that alter membrane lipid composition can indirectly affect curvature and vesicle behavior. Inhibitors of fatty acid synthase (FASN), such as TVB-2640, reduce the production of curvature-promoting unsaturated fatty acids and are currently in clinical trials for several solid tumors. Stearoyl-CoA desaturase (SCD) inhibitors have similar effects and may synergize with PI3K or Ras inhibitors to suppress signaling compartmentalization.

Similarly, statins, originally developed as cholesterol-lowering agents, have been shown to disrupt lipid raft integrity and impair Ras membrane association. Farnesyltransferase inhibitors (FTIs), which block Ras prenylation, further reduce its curvature-dependent membrane anchoring and are undergoing clinical reevaluation, particularly in combination with immunotherapies or MEK inhibitors [90,91].

### 5.4. Diagnostic and Imaging Tools Targeting Curvature

Curvature-targeting diagnostics represent a new dimension in cancer imaging and therapy monitoring. Fluorescent biosensors based on BAR domains or lipid-binding motifs can visualize areas of high membrane curvature in live cells and tissues. These tools provide real-time readouts of membrane architecture and may help identify aggressive tumor phenotypes. Super-resolution microscopy techniques, such as STED and PALM, combined with curvature-specific probes, allow for subcellular resolution of membrane dynamics [92,93]. These technologies could serve as both diagnostics and tools for evaluating therapeutic efficacy in clinical settings [94,95].

## 6. Challenges and Future Perspectives

There are key conceptual and technical challenges that must be addressed to fully position membrane curvature as a functional and translational axis in cancer biology. A major limitation arises from the intrinsic complexity and redundancy of curvature-regulating mechanisms, where lipids, cytoskeletal forces, and curvature-modulating proteins operate through overlapping and compensatory pathways, making it difficult to isolate dominant regulators or predict system-level outcomes. These challenges are compounded by current limitations in measuring membrane curvature dynamically and quantitatively in living systems, particularly within physiologically relevant tumor contexts.

### 6.1. Mechanistic Complexity and Redundancy

One major challenge is that the regulation of membrane curvature in cancer involves highly redundant and overlapping mechanisms. Multiple curvature-inducing proteins, such as BAR domain family members and dynamin-related proteins, can compensate for one another. Additionally, curvature is influenced by dynamic interplay between biochemical signals and mechanical forces, including those from the cytoskeleton and extracellular matrix, which further adds to its context-dependence. Similarly, lipids with distinct shapes can collectively maintain curvature dynamics even when specific lipid species are depleted. This redundancy obviously complicates therapeutic targeting [96].

### 6.2. Real-Time Measurement of Curvature in Living Systems

Accurately measuring membrane curvature in living systems remains another major challenge. Although super-resolution microscopy techniques like STED, PALM, and cryo-electron tomography have advanced our ability to visualize nanometer-scale curvature, their application in live or in vivo systems is still limited. Developing genetically encoded curvature biosensors and integrating them into advanced imaging platforms could allow real-time, high-resolution mapping of curvature changes in tumor environments [97,98].

### 6.3. Application of Artificial Intelligence and Systems Biology

Machine learning and systems biology approaches are proving increasingly valuable for studying membrane curvature. AI-based image analysis can automatically quantify curvature features from high-resolution microscopy data, correlating them with cancer phenotypes or therapy responses. Integrating multi-omics datasets—such as lipidomics, proteomics, and transcriptomics—can uncover hidden regulatory networks that control curvature-related processes, revealing new targets for intervention [99].

Advanced live-cell imaging, optogenetics, and inducible protein-lipid relocalization tools will be essential for dissecting these relationships. Furthermore, computational modeling of curvature-associated signaling nodes may help predict how changes in membrane architecture affect cell fate decisions.

### 6.4. Organoid and Microfluidic Models for Drug Testing

Traditional 2D culture systems fail to replicate the curvature complexity of tumors in vivo. Organoids and microfluidic “tumor-on-a-chip” systems recreate more realistic curvature dynamics, matrix stiffness gradients, and fluidic conditions. These platforms are ideal for testing drugs that target curvature-modulating pathways or nanoparticles designed to exploit curvature-specific uptake [100].

Multi-omics integration—combining lipidomics, transcriptomics, proteomics, and imaging data—could also reveal hidden networks that control curvature in cancer cells. These datasets can be analyzed using network inference algorithms to discover new drug targets or resistance mechanisms [101].

### 6.5. Translational Potential and Clinical Integration

Integrating curvature-focused diagnostics and therapeutics into clinical practice requires robust validation. Imaging tools and circulating vesicle biomarkers that reflect curvature changes could complement existing diagnostic modalities. Moreover, curvature-targeting nanomedicines should be evaluated in patient-derived xenografts and organoid models to ensure efficacy and specificity before clinical translation [102].

Despite their conceptual appeal, curvature-targeting strategies face important translational limitations that must be carefully considered. Membrane curvature is a fundamental property of all cells, raising concerns about systemic toxicity and on-target effects in normal tissues, particularly those with high membrane plasticity such as immune, epithelial, and neuronal cells. In addition, cancer cells exhibit substantial redundancy and adaptability in curvature-regulating mechanisms, whereby inhibition of a single curvature-modulating protein or pathway may be bypassed through compensatory lipid remodeling, cytoskeletal reorganization, or alternative trafficking routes. A further challenge lies in specificity, as curvature signatures are often quantitative rather than qualitative, differing in magnitude, persistence, and spatial organization between normal and malignant cells. Addressing these limitations will likely require strategies that exploit cancer-specific curvature dependencies rather than global curvature inhibition, including combination therapies that couple curvature perturbation with oncogenic pathway blockade, context-dependent delivery systems such as curvature-responsive nanocarriers, and experimental validation in advanced models including organoids and tumor-on-a-chip platforms. Together, these approaches may help transform membrane curvature from a biophysical vulnerability into a clinically actionable therapeutic axis.

## 7. Cancer Types Most Influenced by Membrane Curvature

The role of membrane curvature is particularly pronounced in cancers with high invasive and metastatic potential. In breast cancer, it is a key driver of EMT and metastasis in breast tumors [4]. Glioblastoma displays aggressive infiltration supported by upregulation of curvature-sensitive proteins, facilitating enhanced migration through brain tissue. Pancreatic cancer, dominated by KRAS mutations, relies on curvature-mediated Ras localization and activation, making membrane dynamics central to its oncogenic signaling. Colorectal cancer features altered lipid metabolism and dysregulated Wnt signaling, both of which are closely tied to curvature mechanisms. In prostate cancer, curvature is linked to increased exosome secretion and metastatic niche formation. These cancers exemplify how biophysical membrane properties integrate with oncogenic pathways to drive progression, invasion, and therapy resistance [103,104].

In conclusion, curvature is more than geometry—it is biology in motion. Embracing its complexity may open new doors in the quest to understand and overcome cancer. However, in order to exploit the opportunities in this area, future research must integrate emerging tools in lipidomics, AI-assisted image analysis, organoid modeling, and CRISPR-based functional screening to build a holistic, real-time map of curvature dynamics in cancer. As interdisciplinary collaboration accelerates, membrane curvature is poised to become a cornerstone of next-generation cancer diagnostics and therapeutics.

## 8. Emerging Tools and Technologies for Studying Membrane Curvature

Beyond optical methods, cryo-electron tomography offers complementary three-dimensional, near-native views of membrane curvature at organellar and subcellular levels, providing ultrastructural insight into curved membranes within endosomes, mitochondria, and the endoplasmic reticulum [105]. While cryo-based techniques are limited in throughput and live-cell applicability, they provide essential structural benchmarks that inform and validate fluorescence-based observations.

In parallel, genetically encoded curvature biosensors and optogenetic tools are increasingly being developed to monitor or manipulate membrane curvature in real time. These tools enable dynamic interrogation of curvature changes during processes such as epithelial–mesenchymal transition, endocytosis, and cell migration, linking membrane architecture directly to functional outcomes [106]. It will be very promising approach if we integrate curvature imaging with lipidomics, proteomics, and computational image analysis, which will allow quantitative mapping of curvature landscapes and their coupling to oncogenic signaling states.

Finally, advanced experimental platforms such as organoids and microfluidic systems provide physiologically relevant contexts in which curvature dynamics can be studied under controlled mechanical and biochemical conditions. These models preserve tissue-level architecture, membrane tension gradients, and signaling heterogeneity, making them well suited for testing curvature-modulating drugs and nanotherapeutics.

Collectively, these emerging tools establish membrane curvature as a measurable, dynamic, and functionally informative parameter, positioning it as a critical biophysical axis for understanding cancer progression and for evaluating therapeutic responses.

## 9. Conclusions

The integration of membrane curvature into the framework of cancer biology represents a paradigm shift in how we understand cellular transformation, invasion, and therapy resistance. By connecting mechanical properties of the membrane to molecular signaling networks, researchers can uncover new layers of oncogenic regulation that have previously gone unnoticed. As our tools for probing and manipulating membrane curvature become more sophisticated, the field is likely to uncover novel biomarkers, drug targets, and therapeutic strategies. Importantly, membrane curvature intersects with key biological systems beyond cancer, including neurodegeneration and immune signaling, indicating broad relevance. Interdisciplinary collaboration across cell biology, physics, engineering, and clinical oncology will be essential to realize the full therapeutic potential of targeting membrane curvature.

Table 1 summarizes the principal curvature determinants, their biophysical effects, the resulting impact on protein organization, associated signaling pathways, and corresponding cancer phenotypes.

## Figures and Tables

**Table 1:** Integrative Framework Linking Membrane Curvature to Cancer Signaling and Phenotypes.

Curvature Determinant	Biophysical Effect	Impact on Protein Organization	Key Signaling Pathways	Cancer Phenotypes	References
Altered lipid composition (↑ cone-shaped lipids, PIP2, cholesterol)	Spontaneous curvature; altered membrane stiffness	Ras nanoclustering; recruitment of curvature-sensitive proteins	Ras/MAPK, PI3K/AKT	Sustained proliferation, metabolic rewiring	[15,58,67,70]
BAR/F-BAR/ENTH proteins (e.g., CIP4, amphiphysin)	Scaffolding and membrane bending	Assembly of signaling hubs at endocytic pits	Rac1, Cdc42, Ras	Migration, invasion, metastasis	[20,21,26,59,75]
Actin dynamics & membrane tension	Force-driven membrane deformation	Stabilization of protrusive structures and signaling microdomains	Ras, YAP/TAZ, TGFβ	EMT, mechanotransduction, invasion	[35,36,38,62]
Curvature-dependent Ras relocalization (e.g., TGFβ-induced)	Positive curvature enhances membrane residency	Increased Ras activation and nanocluster stability	Ras/MAPK–SMAD feedback	EMT, metastatic progression	[4]

## Data Availability

No new data were created or analyzed in this study.
